# Fungal genotype determines survival of *Drosophila melanogaster* when competing with *Aspergillus nidulans*

**DOI:** 10.1371/journal.pone.0190543

**Published:** 2018-01-02

**Authors:** Annika Regulin, Frank Kempken

**Affiliations:** Botanisches Institut und Botanischer Garten, Christian-Albrechts-Universität, Kiel, Germany; Universidade de Sao Paulo, BRAZIL

## Abstract

Fungi produce an astonishing variety of secondary metabolites, some of which belong to the most toxic compounds in the living world. Several fungal metabolites have anti-insecticidal properties which may yield advantages to the fungus in competition with insects for exploitation of environmental resources. Using the *Drosophila melanogaster/Aspergillus nidulans* ecological model system to assess secondary metabolite mutant genotypes, we find a major role for the *veA* allele in insect/fungal confrontations that exceeds the influence of other factors such as LaeA. VeA along with LaeA is a member of a transcriptional complex governing secondary metabolism in *A*. *nidulans*. However, historically a mutant *veA* allele, *veA1* reduced in secondary metabolite output, has been used in many studies of this model organism. To test the significance of this allele in our system, *Aspergillus nidulans veA* wild type, *veA1*, *ΔveA* and *ΔlaeA* were evaluated in confrontation assays to analyze egg laying activity, and the survival rate of larvae. The *veA1* genetic background led to a significant increase of larval survival. Adult flies were observed almost exclusively on *veA1*, *ΔveA* or *ΔlaeA* genetic backgrounds, suggesting a role for the velvet complex in insect/fungal interactions. This effect was most profound using the *veA1* mutant. Hence, larval survival in confrontations is highly affected by the fungal genotype.

## Introduction

Fungi and animals interact in many ways, including mutualistic, predatory, pathogenic, or competitive interrelationships [1,2], which so far are only poorly understood. With respect to the tremendous importance of fungi for many terrestrial ecosystems [3], a better knowledge of the different types of interactions of fungi with animals, their causes and consequences is of ecological importance. Fungivores including insects, nematodes, mites, and others have a tremendous effect on fungi in soil ecosystems [4]. In addition, saprotrophic fungi, which exploit food sources such as fruit or carrion are engaged in competitive interactions with animals living on the same resources [1,5]. In both circumstances fungivores and competitors can harm fungi seriously and thus may affect negatively fungal evolutionary fitness [6–8].

Fungi have developed several lines of defense against animal antagonists including fungivores and competitors [9]. Filamentous fungi possess an inducible resistance based on fruiting body lectins, which are specific for glycans of fungivores, including insects [10,11]. Fruiting body lectins are small, water-soluble molecules in the cytoplasm of fruiting body hyphae [12]. Likewise, trypsin-specific protease inhibitors from the basidiomycetes *Clitocybe nebularis* have defensive functions, and similar expression patterns and subcellular locations as the fruiting body lectins [13]. Finally, toxic fungal secondary metabolites (SMs) provide an extensive repertoire in filamentous fungi, some of which exhibit insecticidal activities such as aflatoxin, sterigmatocystin (ST) and bassianolide [14–16].

Fungal secondary metabolite genes are organized in gene clusters [17], and synthesis and regulation of several mycotoxins have been studied in much detail [18]. In addition to cluster-specific transcription factors, global regulators of secondary metabolism have been identified such as the *Aspergillus nidulans* LaeA protein, which together with VeA and VelB forms the conserved velvet complex [19]. The velvet complex is required for secondary metabolite production in filamentous fungi [20]. Orthologues of all three proteins occur in many fungal species [21–27] and many studies have shown that deletion of either *laeA* or *veA* results in a general decrease in production of secondary metabolism [20,28–30]. However, the null strains are not equivalent in regulatory effects on secondary metabolite synthesis and, at least in *A*. *nidulans*, in *ΔveA* the cryptic orsellinic acid gene cluster is up-regulated [31] and *veA* is reported as a repressor of penicillin synthesis [32].

The VeA protein plays a major role in activating sexual development and inhibiting asexual development. VeA is a member of the family of *velvet* proteins that also includes VelB, VelC and VosA. These *velvet* regulators are found in many Ascomycetes [33] and appear to play a crucial role in regulating fungal development [28,34,35]. This includes the mode of reproduction and balancing the occurrence of both types of reproductive spores, i.e. conidia and ascospores [36–39]. VeA, which interacts with VelB, is essential for the activation of sexual reproduction and indirectly inhibits conidia formation [19,30,40,41]. The participation of VeA as the main regulator in different processes, also beyond the velvet complex, is probably due to the spatially and temporally controlled specific protein-protein interaction with other regulators. VeA interacts with several other proteins including phytochrome FphA [42] and LaeA [43,44].

In *A*. *nidulans*, the *veA* gene can be present in different allelic forms. The wild type *veA* gene encodes a 573 amino acid protein with highly conserved domains (*velvet* domains and nuclear localization signal in its N-terminus [45]. The *veA1* mutant allele which is used in this study, has a point mutation from G-to-T at position +3 mutating the start codon ATG to ATT. Hence, a second ATG codon (amino acid 37 in the *veA*^*+*^ allele) is used for translation of the mutant allele leading to a truncation of 36 amino acids at the N-terminus [40]. Consequently, the *velvet* domain is partially truncated and the NLS is shortened by half. An intact N-terminus is required for a successful interaction of VeA with VelB to complete the heterotrimeric complex including LaeA to contribute to secondary metabolite production. Hence the formation of the assembled velvet complex of VeA, VelB and LaeA, as well as secondary metabolite production is strongly reduced in the *veA1* background [19,36]. However, prior to the knowledge of the *veA1* allele and how it impacts function of the velvet complex, this allele was used extensively in *A*. *nidulans* research due to the fact that it was easier to harvest asexual spores from this strain since its discovery in 1965 [28,46].

We thus became interested to assess the impact of the velvet complex, specifically three *veA* alleles, on *Aspergillus* insect interactions. Previous studies have assessed the influence of LaeA on *Aspergilus/*insect confrontations both in competition with the fruit fly *Drosophila melanogaster* [47,48] and in defense against the springtail *Folsomia candida* [49]. However, no particular studies have been made with the direct focus of comparing the wild type *veA* strain to either a *veA1* or *ΔveA* (e.g. loss of *veA*) in confrontations with insects. In this study, we assess effect of these three alleles and specific mutants of some secondary metabolite gene clusters on the egg laying activity and survival of *Drosophila melanogaster* under conditions competing with *A*. *nidulans*. An earlier study examined whether adult *Drosophila* females avoid laying eggs on mould-infested substrates using the *veA1* strain as the wild type [47]. The authors could not observe an effect of the fungus on the number of eggs the females laid and concluded that *Drosophila* females did not avoid *A*. *nidulans* patches when laying eggs. In contrast, here we find *Drosophila* females do avoid laying eggs on several strains of *A*. *nidulans*, particularly in case of confrontation with the *veA*^*+*^ wild type. These findings suggest that in confrontation experiments with *Drosophila* and *Aspergillus* the choice of the strain to be used is of crucial importance.

## Results and discussion

**Fig 1:**
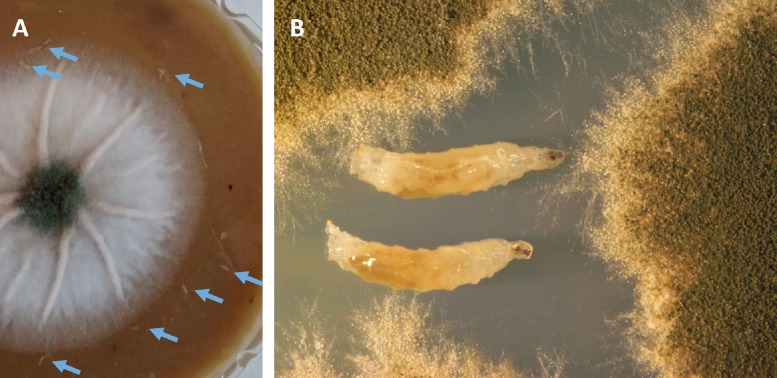
Fungal insect competition; pictures from experimental setup. **(A)** *A*. *nidulans* colony with insect larvae (arrows) **(B)** Close-up of a fungi-insect-confrontation.

We set out to analyze any effect of wild type *A*. *nidulans* compared to a variety of mutants under competing conditions on egg-laying activity, larval survival, and number of adult flies observed. The experimental set-up is shown in Fig 1. The mutant strains we used are shown in Table 1. This includes the *veA*^*+*^ wild type, as well as the *veA1*, Δ*veA* and Δ*laeA* strains. In addition, we employed some deletions of specific secondary metabolite genes to compare the effect of global regulation to the loss of individual SMs. Δ*stcJ* (sterigmatocystin mutant), Δ*mdpG* (monodictyphenone mutant), Δ*aptA* (asperthecin mutant), Δ*aptB* (asperthecin mutant), Δ*aptC* (asperthecin mutant) and a mutant lacking the entire sterigmatocystin cluster (Δ*ST*). Sterigmatocystin (ST) has been shown to exhibit anti-feeding behavior in previous studies [16,50,51] whereas monodicytyphenone [52,53] and asperthecin [54] mutants have not been assessed in insect studies previously.

**Table 1:** Fungal strains used.

Strain	Genotype	Source or Reference
FGSC A4	Glasgow wild type (*veA*^+^)	Fungal Genetic Stock Center
FGSC A752	*pabaA1*, *sB43*, *amdI66 amdS1005*, *alcR125 amdA7*,*wild type veA1*	Fungal Genetic Stock Center
RJW41.A	*ΔlaeA*::*metG; metG1*, *veA+*	[19]
RSCS4	*ΔstcJ*::*argB*, *veA+*	[65]
RJMP103.5	*veA*^*+*^, WT	[59]
RAAS146.186	*ΔST* cluster	[53]
RAAS146.3	*ΔmdpG*::*pyroA*, *veA+*	[53]
RJW112.2	*ΔveA*::*argB*, *veA+*	[19]
RJMP240.8	*ΔaptA*::*pyroA*, *veA+*	[54]
RJMP238.8	*ΔaptB*::*pyroA*, *veA+*	[54]
RJMP239.7	*ΔaptC*::*pyroA*, *veA+*	[54]

**Fig 2:**
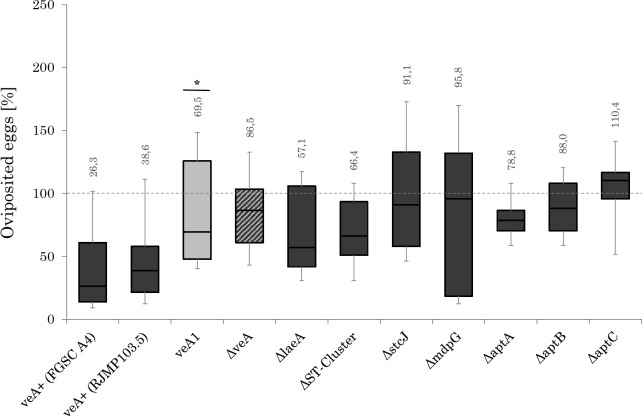
Egg laying activity of adult *Drosophila melanogaster* in presence of various *Aspergillus nidulans* strains after 24 h. On the ordinate, the number of deposited eggs is shown as a relative frequency and refers to the fungus-free control. On the abscissa the different fungal strains are outlined. The fungal strains are listed in Table 1. 24-well-plates were filled with 2 ml of a combination of *Drosophila* corn meal medium and fungal glucose minimal medium (DPM/GMM). For generating an equal size of colony conidia were transferred from fungi culture in each well by stamping with a pestle. After 6 h fungal pre-growth one fertilized female *Drosophila melanogaster* of the same generation was added in each well. After 24 h at 30°C in darkness the vinegar flies have been removed and the deposited eggs were enumerated. For each confronted colony and for mold-free control N = 8. Fungal strains colored with dark grey boxes possess a *veA*^*+*^ genetic background whereas the light grey boxes represent fungal strains harboring the *veA1* mutation. The shaded area box displays a *ΔveA* mutant which carries due to the entire deletion of *veA* neither a *veA*^*+*^ nor the *veA1* mutation.

Fig 2 gives the results for egg laying behavior of imagos during competition. Egg laying activity on medium without a competitor was utilized as control and was set to 100%. Egg laying activity on medium with a fungal mycelium was lowest when competing with the wild types *A*. *nidulans* (FGSC A4 and RJMP103.5) with medians of 26.3% to 38.6%. On the *veA1*, Δ*veA*, Δ*laeA*, *ΔST-cluster* and Δ*aptA* mutants egg laying activity was somewhat higher with medians of 57.1% (Δ*laeA)* and highest of 88.0% (Δ*aptB)*. The difference in egg laying activity between *veA1* and wild type is highly significant (p < 0.05). Highest egg laying activity was observed for the Δ*stcJ* mutant (91.9%), *ΔmdpG* (95.8%) and *ΔaptC* (110.4%) in a *veA*^*+*^ genetic background.

**Fig 3:**
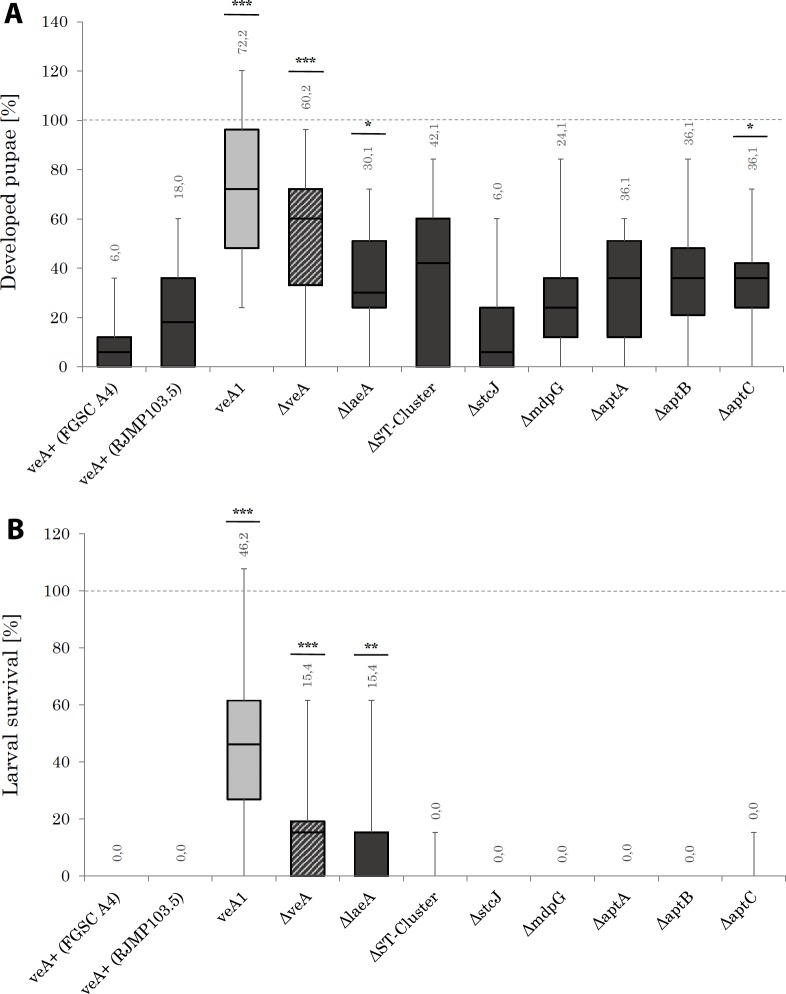
**(A)** *Drosophila melanogaster* development form larva to pupa during confrontation with different *Aspergillus nidulans* strains. On the ordinate, the number of developed larvae is shown as a relative frequency and refers to the mould-free control. On the abscissa the different fungal strains are outlined. The fungal strains are listed in Table 1. 2 ml microtubes were filled with 1 ml of medium as described in Fig 2. Transfer of respective *Aspergillus* conidia in tubes was performed by using a pestle. Tubes were closed with cotton plugs. After incubation for 14 h at 30°C in darkness conidia were confronted with ten sterile 1^st^ instar larvae. The experimental setup was incubated at 30°C under dark condition in a humid chamber. The experiment was terminated when no further development could be observed and developed pupae were enumerated. Fungal strains colored with dark grey boxes possess a *veA*^*+*^ genetic background whereas the light grey boxes represent fungal strains containing the *veA1* mutation. The shaded area box displays a *ΔveA* mutant, which carries neither a *veA*^*+*^ nor the *veA1* mutation since the entire lack of the gene. For each treatment and for mold-free control N = 16. **(B)** Larval survival and imaginal viability of adult *Drosophila melanogaster* under fungal exposure. On the ordinate, the number of living flies is shown as a relative frequency and refers to the mould-free control. On the abscissa, the different fungal strains are outlined. The fungal strains are listed in Table 1. 2 ml mircotubes were filled with 1 ml of medium as described in Fig 2. Transfer of respective *Aspergillus* conidia in tubes was performed by using a pestle. Tubes were closed with cotton plugs. After incubation for 14 h at 30°C in darkness conidia were confronted with ten sterile 1^st^ instar larvae. Subsequent incubation occurred at 30°C under exclusion from light in a humid chamber. Fungal strains colored with dark grey boxes possess a *veA*^*+*^ genetic background whereas the light grey boxes represent fungal strains containing a *veA1* point mutation. The shaded area box displays a *ΔveA* mutant which carries neither a *veA*^*+*^ nor a *veA1* point mutation since the entire lack of the gene. Experimental units were checked every day at the same time point whether flies have hatched. Hatched flies were removed and enumerated. For each treatment and for mold-free control N = 16.

For analysis of larval survival in each experiment ten larvae of the first instar (L1) were used. Larval survival was measured by counting the number of developed pupae after twelve days. Results for larval survival are given in Fig 3. A. As a control, 10 larvae were placed in the mould-free experimental units. The presence of wild type *A*. *nidulans* (FGSC A4) significantly reduces larval survival, as has been shown earlier [1,6,7]. The largest number of pupae was observed with the *veA1* strain, with a very high significance compared to the wild type (p < 0.001), whereas on the Δ*veA* strain fewer pupae (also p < 0.001) were observed (medians of 72.2% vs. 60.2%). The Δ*stcJ* mutant showed a similar effect on larvae with both wild types. Interestingly, there is a strong difference in the median of the Δ*stcJ* mutant compared to the strain with the entire sterigmatocystin cluster deleted (medians of 6.0 vs. 42.1%). Apparently, the lack of the entire cluster strongly increases insect survival. As the Δ*stcJ* mutant blocks sterigmatocystin production at a very early step the reduced larval survival may be due to the fact that other enzymes encoded by the sterigmatocystin cluster may still be active and using polyketide precursors present in the cell. Specifically, we note that StcJ is a fatty acid synthase subunit involved in sterigmatocystin synthesis and sterigmatocystin can be restored to some degree in the Δ*stcJ* background, presumably by fatty acid availability in the environment [55].

The three asperthecin mutants had a median of 36.1% which is significant (p < 0.05) in the case of the *ΔaptC* mutant. Interestingly, the Δ*laeA* mutant, which is globally impaired in secondary metabolite production [20], in other studies has been shown to beneficial to larval survival [47]. However, in our experiments, less pupae developed on Δ*laeA* than on the *veA1* or Δ*veA* strains (medians of 30.1% vs. 72.2% or 60.2%). This result is highly significant (p < 0.001). We point out that there is a significant difference (p < 0.05) between the wild type *veA*^*+*^ and the Δ*laeA* mutant (6% or 18% vs. 30.1%), suggesting that the reduction of secondary metabolites production in the *laeA* mutant is beneficial for pupae development in *Drosophila*. Admittedly, this role seems to be somewhat less substantial than that of *veA* (72.2% or 60.2% vs. 30.1%). Hence, both parts of the velvet complex seem to produce secondary metabolites to defend against competing insects. Finally, using the Δ*mdpG* mutant we observed a median of 24.1% of developed pupae.

In a last set of experiments, we analyzed the number of adult flies to emerge after competition (Fig 3B). Only on the *veA1*, Δ*veA* and Δ*laeA* mutants were a considerable number of adult flies was observed, despite the fact that large numbers of up to 60% (median) developed pupae were also found on other mutants. The highest number of adult flies (median of 46.2%) was identified on the *veA1* mutant. The same significance level (p < 0.001) was determined in case of Δ*veA* (median of 15%). For the Δ*laeA* mutant significance was still high (p < 0.01). In case of the ΔST-cluster and Δ*aptC* mutants the error bars display positive values due to single negligible hatched individuals during the conducted experiments. Nonetheless, the numbers of adult flies featured medians of 0%. Apparently, the deletion of the ST-cluster or the *aptC* gene has no effect on Drosophila survival.

Furthermore, we examined the time-based eclosion behavior of the living imagos, which were exposed to the different *A*. *nidulans* strains shown in Table 1. Data sets were obtained from these survival experiments and represent means of 16 independent experiments. The numbers of hatched adults are given in percentage of the mold-free control. Irrespective of the fungal strain used, the majority of all the flies started to hatch out from their puparia at the ninth day of the confrontation. The last few imagos were eclosed at day ten. This finding does not correspond with the general life cycle of wild type *D*. *melanogaster*. The complete development from deposited egg to adult imago lasts around ten days at a temperature of 25°C [56]. In our experiments, however, we used 30°C as the incubation temperature. According to the Bloomington Drosophila Stock Center at a temperature of 29°C the imagos usually slip on the seventh day (BDSC, http://flystocks.bio.indiana.edu/Fly_Work/culturing.htm). Hence it appears that larval development and the final eclosion are delayed due to confrontation with different strains of *A*. *nidulans*.

All of our findings demonstrate a strong relevance of different *veA* alleles for egg laying, larval survival, and development of adult flies. Based on our study, all further fungal-insect competition experiments should be done using the *veA*^*+*^ background as use of the *veA1* background is likely to compromise the interpretation of results. The *veA1* allele results in reduced functioning of the velvet complex and concomitant reduction in secondary metabolite production [19,36] which likely contributes to the results we observed in our study. A recent transcriptome analysis revealed that about 26% of protein coding genes were differentially regulated in a *ΔveA* mutant compared to wild type *Aspergillus nidulans* [26] thus demonstrating the strong influence of the *veA* gene.

Both *ΔveA* and *ΔlaeA* strains exhibit the same effect on the tested properties of *Drosophilae* (15% of hatched imagos), which indicates the important role of the velvet complex during a fungus-insect-interaction. For example, microarray analyses have revealed a variety of genes involved that are under the control of LaeA [29,57,58]. The formation of the heterotrimeric velvet complex is also abolished by deletion of *veA* or reduced in the *veA1* mutant. Like LaeA, transcriptome analysis shows that VeA regulates a large portion of gene expression in *A*. *nidulans* including but not limited to secondary metabolism [26]. It is not surprising that the interaction with *Drosophilae* and *veA1* versus *veA* varies. Transcriptome analysis has shown that 2351 genes, 1549 up-regulated and 802 down-regulated, are differentially regulated in veA vs *veA1* versus *veA* [35]. Furthermore, there could be significant effects on the property of survival of *Drosophilae* due to altered post-translational modifications induced by a *veA1* mutation. For example, the methyltransferase LlmF is capable of interacting with VeA, but not with the VeA1-truncated polypeptide. Hence, LlmF-mediated control of secondary metabolism, especially that of ST, and development, do not occur in a *veA1* background [59–61].

In conclusion, larval survival toward development of adult flies was only observed using the *veA1*, *ΔveA* and *ΔlaeA* strains. These strains also yielded the largest number of developed pupae. All other strains completely prohibited larval survival. Apparently, the velvet complex seems to be an essential component in competition between *A*. *nidulans* and *D*. *melanogaster*.

## Materials and methods

### Fungal strains and growth conditions

*Aspergillus nidulans* strains used in this study are listed in Table 1 and possess either a veA^+^ or a *veA1* background. All strains are stored as glycerol stocks and were grown on glucose minimal medium including supplements if required (GMM) [62] and incubated at 37°C. For *veA1* (FGSC A752) 5 μM para-aminobenzoic acid was supplemented to the GMM.

### Cultivation of Drosophila melanogaster

*Drosophila melanogaster* wild type strain Oregon-C-R (Bloomington Drosophila Stock Center) was reared on DZM at RT with a 12 h:12 h light-dark cycle. DZM contains 6.25% cornmeal (health food store), 6.25% brewer’s yeast, 2% Glucose and 1% agar. In the slightly warmed medium 3% sugar-cane molasses and 3% sugar beet syrup (health food store) were added and boiled up for 15 min. After autoclaving 1% [v/v] 10% propionic acid and 3% [v/v] 10% methylparaben were supplemented.

### Dechorionation of Drosophila embryos

Experiments were initiated with eggs deposited overnight on apple juice agar (3% agar in 70% H_2_O, after autoclaving add 30% apple juice, 0.05% methylparaben, 2% ethanol) by 5 to 7 days old mated females. The eggs were washed with sterile water from the agar by use of a brush. The water containing the eggs was filtered through a funnel lined with gauze (150 μm). This gauze was tilted gently and incubated in a 6% sodium hypochlorite solution for 15 min. Afterwards the water was filtered through fresh gauze and dechorionated eggs were washed twice with 70% ethanol and sterile water. The gauze containing the eggs was transferred on methyparaben agar (3% [v/v] 10% methyparaben, 3% agar) and the dechorionated eggs were moved on DZM using a small brush. Eggs were cultured at 25°C and night-day-rhythm until they were used for further experiments.

### Method to generate reproducible colony sizes

For a verifiable and meaningful analytical evaluation of all results, all investigations should be based on constantly reproducible colony sizes, which are independent of the *A*. *nidulans* strain used. To test the optimal technique, conidia were transferred to the medium in two different ways. S1 Fig shows examples of both methods. In current research the conventional suspension technique is used as the classical method [47,48,63]. In this case, a defined number of conidia is transferred to the medium in a certain solvent volume, but the solution spreads differently and the conidia can float unevenly (Panel A in S1 Fig). When using a small-scale system, the size of the growth surface of the spores with respect to the number and size of conidia depends primarily on the distribution of the conidia on the medium and not on the number of plated spores. By dispensing the conidia suspension, the solvent is spread over the medium to different extents. Consequently, the solution containing the conidia also float differently. If the spores begin to germinate, this results in correspondingly various large colonies. To circumvent this effect, the conidia were stamped by means of a pestle from a desired colony on the confrontation medium, whereby an equal area is always inoculated (conical pestle, dimensions: total length 70 mm, shaft Ø 4,9 mm, cone narrowed from 6 to 2.5 mm, keel length 10 mm, Roth company) (Panel B in S1 Fig).

In order to quantitatively analyze the different growth effects of the fungus using either technique and to prove the efficiency of the developed method independently on the different strains and mutants, the colonies of the diverse *A*. *nidulans* strains and mutants were measured. The values were plotted graphically, after the conidia were transferred as described above in two different ways (S2 Fig).

In case of the common suspension method 1 μl of a suspension containing 1000 conidia of the selected strain were transferred to the medium by placing the droplets onto the substrate surface [47] (n = 11) (Panel A in S2 Fig). For the stamping technique, the pestle was pressed centrally into the selected colony and the conidia adhering to it were transferred to the confrontation medium by stamping. This procedure was performed with each of the strains and mutants used in this study (n = 15) (Panel B in S2 Fig). After inoculation of conidia, the experimental unites were incubated for 48 h at 30°C under dark conditions. Subsequently, the grown colonies were measured.

The stamping method produces constant size ratios independent of the strain used. The detected deviations on the colony diameters arise not due to potential differences in the growth of the different fungal strains by genotype, which is the decisive methodological advantage of the stamp method established here.

### Statistical procedure

Since the obtained data from the development and survival experiments do not correspond to the Gaussian normal distribution, the box plot representation was used to illustrate these results. For a better comparability of the results among each other, this type of representation was also used for the oviposition experiments.

In contrast to the mean value with the standard error bars in a lane plot, the median with boxplot proves to be the best mathematically and statistically best and correct variant, especially for non-normalized values. In addition, the boxplot provides much more information about the actual distribution of the data, which is of great importance in ecological experiments due to the wide range of variations [64].

Statistic significances of each experiment were calculated by using the D‘Agostino & Pearson omnibus normality test for determining the Gaussian distribution of the measured datasets. For One Way ANOVA the Dunnett’s Multiple Comparison Test was executed in case of the egg laying activity analysis.

The statistical significance of the datasets for the larval development and the adult survival study, the Kruskal-Wallis Test as One Way ANOVA was used and in particular, the Dunn’s Multiple Comparison Test was executed.

### Egg laying activity

2 ml of a combination of DPM and GMM (6,25% cornmeal, 6,25% brewer’s yeast, 2% glucose, 4% sucrose, 1,2% 1 M phosphate buffer, 2% 50× MM salts, 0,1% 1000× Hutner’s trace elements, 1% agar, boiled up for 15 min and autoclaved at 112°C. For phosphate buffer, 50× MM salts and Hutner’s trace elements see (http://www.fgsc.net/Aspergillus/asperghome.html) were filled in 24-well plates and pre-incubated overnight at 30°C. An equal size of colony was generated by transferring the conidia from an appropriate colony to the center of the well employing a pestle. After 6 h growth at 30°C in darkness one five days old fertilized female of the same generation was placed to each grown colony in the well. Additionally, one single fertilized female was set on mold-free DPM/GMM as a control. The interaction was stopped after 24 h. The imagos were removed and the deposited eggs were enumerated under the binocular.

### Larval survival and development

2 ml eppendorf tubes were filled with 1 ml of DPM/GMM (see egg laying activity), closed with cotton plugs and pre-incubated overnight at 30°C. The transfer of *Aspergillus* conidia into these tubes was performed by using a pestle. After incubation for 14 h at 30°C in darkness conidia were confronted with ten sterile 1^st^ instar larvae which were washed once with 1× PBS before applied. Mold-free sample was used as a control. The experimental setup was incubated at 30°C under dark condition in a humid chamber. Every 24 h the number of the hatched imagos were recorded and removed. The experiment was terminated when no further eclosion and development could be observed. Finally, the developed pupae were enumerated.

## Supporting information

S1 Fig Generating equal sized fungal colonies. **(A)** Transfer of *A*. *nidulans* wild type (*veA*^*+*^) conidia by using the suspension technique. For each replicate 1 μl of a suspension containing 1000 conidia was dropped on the surface of the medium. Incubation of spores was performed for 24 h at 30°C in the dark. (B) Stamped *A*. *nidulans* wild type (*veA*^*+*^) conidia by using a pestle. Incubation of spores was performed for 24 h at 30°C in the dark.
(PDF)

S2 Fig Growth determinations of the fungal colonies. **(A)** Using the suspension technique: 1 μl of a suspension containing 1000 conidia of the respective fungal strain was dropped on medium (N = 11). Incubation of the conidia took place for 24 h at 30°C in the dark. The ordinate shows the diameter of the respective colonies as the mean value. For a simplified comparability of the different quantities, the weighted arithmetic mean is shown as a grey dashed line and the total mean value of 0.38 mm is given. (**B)** Using the stamping technique. Using a pestle, the conidia of the respective fungal strain were transferred to the medium (N = 15). Incubation of the conidia took place in the dark at 30°C for 24h. The ordinate shows the diameter of the respective colonies as the mean value. For a simplified comparability of the different quantities, the weighted arithmetic mean is shown as a grey dashed line and the total mean value of 0.42 mm is given.
(PDF)
