# Supplementary figures and images for: Fungal genotype determines survival of Drosophila melanogaster when competing with Aspergillus nidulans
Source: PLoS One. 2018 Jan 2;13(1):e0190543. doi: 10.1371/journal.pone.0190543 (PMC5749846; doi:10.1371/journal.pone.0190543)

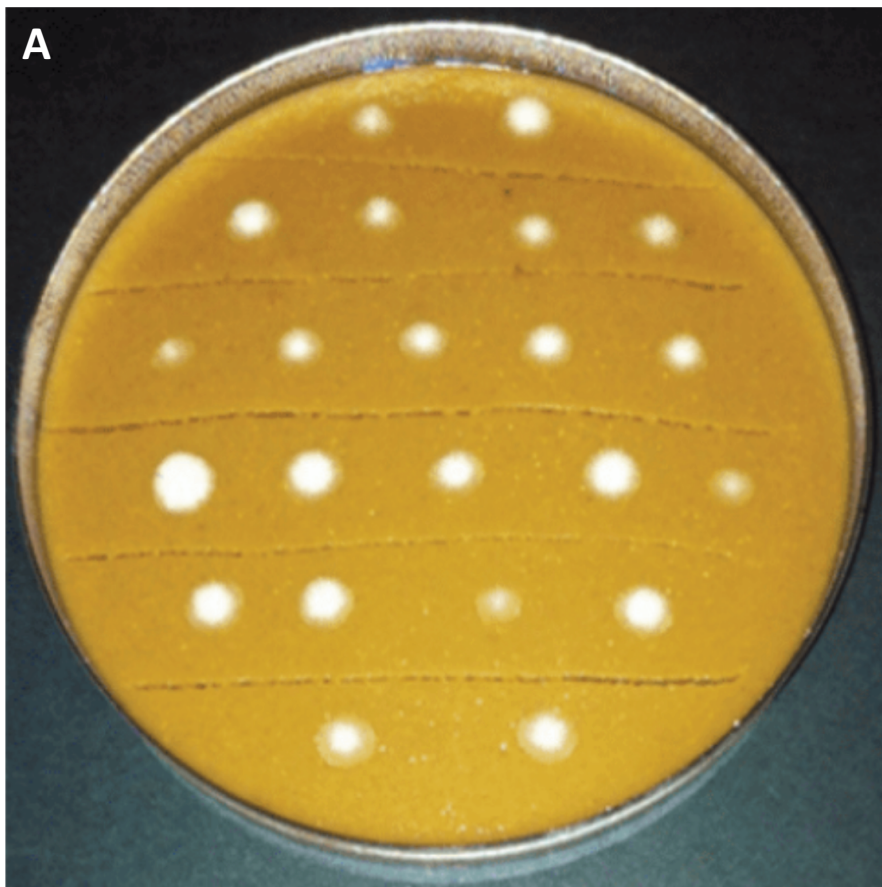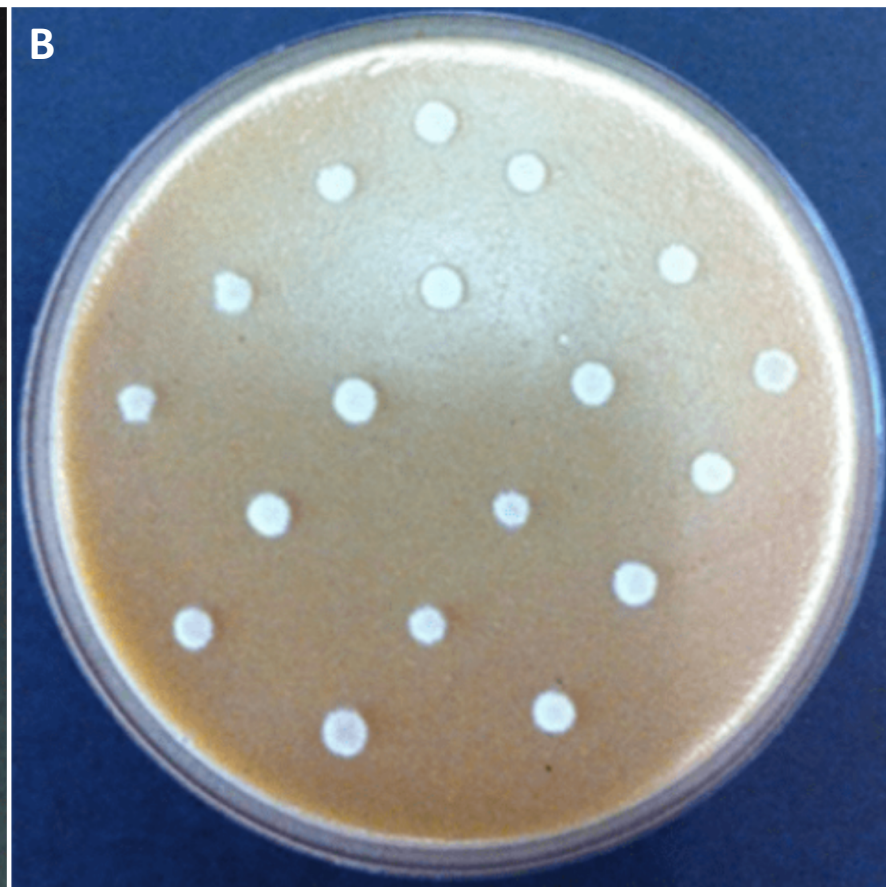

Supplement: S1 Fig — (A) Transfer of A. nidulans wild type (veA+) conidia by using the suspension technique. For each replicate 1 μl of a suspension containing 1000 conidia was dropped on the surface of the medium. Incubation of spores was performed for 24 h at 30°C in the dark. (B) Stamped A. nidulans wild type (veA+) conidia by using a pestle. Incubation of spores was performed for 24 h at 30°C in the dark. (PDF) [file pone.0190543.s001.pdf]

**A**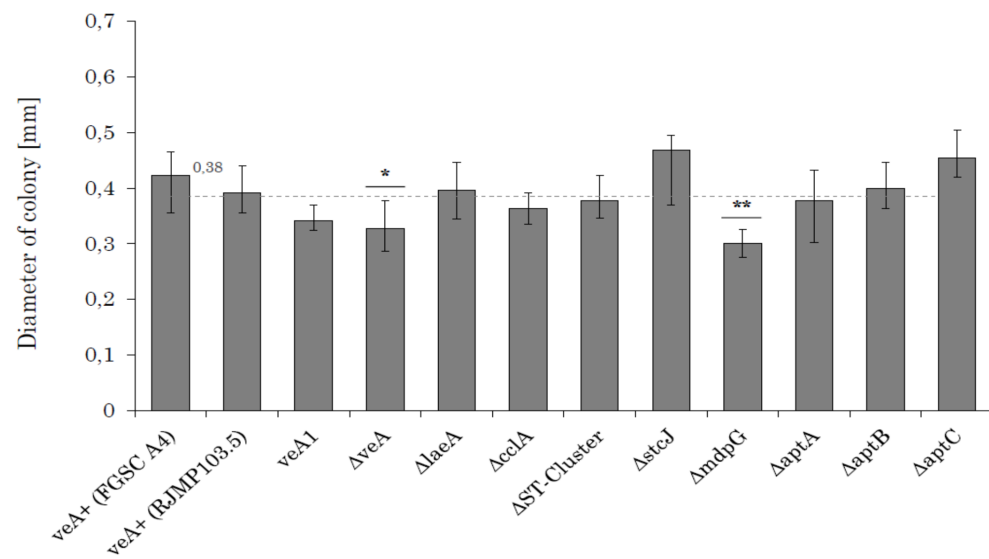**B**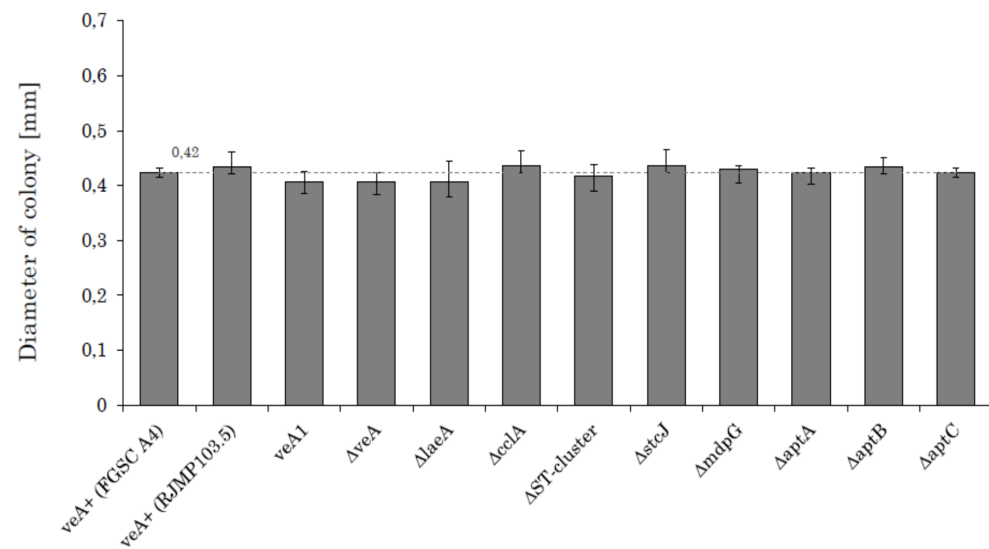

Supplement: S2 Fig — (A) Using the suspension technique: 1 μl of a suspension containing 1000 conidia of the respective fungal strain was dropped on medium (N = 11). Incubation of the conidia took place for 24 h at 30°C in the dark. The ordinate shows the diameter of the respective colonies as the mean value. For a simplified comparability of the different quantities, the weighted arithmetic mean is shown as a grey dashed line and the total mean value of 0.38 mm is given. (B) Using the stamping technique. Using a pestle, the conidia of the respective fungal strain were transferred to the medium (N = 15). Incubation of the conidia took place in the dark at 30°C for 24h. The ordinate shows the diameter of the respective colonies as the mean value. For a simplified comparability of the different quantities, the weighted arithmetic mean is shown as a grey dashed line and the total mean value of 0.42 mm is given. (PDF) [file pone.0190543.s002.pdf]
